# Integrative Approaches to the Treatment of Cancer

**DOI:** 10.3390/cancers14235933

**Published:** 2022-11-30

**Authors:** Kylie O’Brien, Karin Ried, Taufiq Binjemain, Avni Sali

**Affiliations:** 1 NICM Health Research Institute, Western Sydney University, Westmead, NSW 2145, Australia; 2 National Institute of Integrative Medicine, Hawthorn, VIC 3122, Australia

**Keywords:** cancer, integrative medicine, nutritional medicine

## Abstract

**Simple Summary:**

A significant proportion of people with cancer use forms of complementary medicine. Many factors contribute to cancer including some which are modifiable by the patient, such as stress, poor nutrition, vitamin D deficiency, poor sleep and lack of physical activity. This paper discusses why it is important that oncologists help cancer patients to address such factors, and why an integrative approach which combines evidence-based complementary medicines or therapies with orthodox treatment might lead to better outcomes for cancer patients.

**Abstract:**

A significant proportion of cancer patients use forms of complementary medicine or therapies. An integrative approach to cancer management combines conventional medicine with evidence-based complementary medicines/therapies and lifestyle interventions, for the treatment and prevention of disease and the optimisation of health. Its basis is a holistic one; to treat the whole person, not just the disease. It makes use of adjunct technologies which may assist the clinician in diagnosis of early carcinogenesis and monitoring of treatment effectiveness. Many factors contribute to the development of cancer including some which are largely modifiable by the patient and which oncologists may be in a position to advise on, such as stress, poor nutrition, lack of physical activity, poor sleep, and Vitamin D deficiency. An integrative approach to addressing these factors may contribute to better overall health of the patient and better outcomes. Evidence-based complementary medicine approaches include the use of supplements, herbal medicine, various practices that reduce stress, and physical therapies. Individualised to the patient, these can also help address the symptoms and signs associated with cancer and its orthodox treatment.

## 1. Introduction

Cancer is a chronic, systemic illness with a variety of aetiological factors including genetic susceptibility, environmental, and epigenetic factors. Other associated factors include those that are largely modifiable by the individual, including stress, poor nutrition/diet, lack of physical activity, poor sleep, and vitamin D deficiency. Cancer and its orthodox treatment are associated with many symptoms and signs, many of which decrease the ability of patients to complete treatment, such as cancer-related pain, chemotherapy-induced peripheral neuropathy, oral mucositis, anxiety, depression, and poor sleep [1]. Many of these are not well managed with orthodox medicine approaches.

An integrative approach to the treatment of illness like cancer is one in which combines conventional medicine with evidence-based complementary medicines/therapies, nutritional medicine and lifestyle interventions for the treatment and prevention of disease and the optimisation of health. Its basis is a holistic one; to treat the whole person, not just the disease. It makes use of adjunct technologies which may assist the clinician in diagnosis of early carcinogenesis and monitoring of treatment effectiveness, such as circulating tumour cell tests [2,3,4,5]. Therapies that are adjunct to orthodox treatment can, importantly, help the cancer patient deal with many of the symptoms and signs associated with cancer and its orthodox treatment. 

Indeed, there is much evidence to indicate that people with cancer and cancer survivors are using complementary medicines/therapies [6]. Proportions of cancer patients found to use complementary medicines/therapies range from around 40% in some studies to 84% in others [7,8,9,10,11].

An effective integrative approach facilitates a collaborative relationship between practitioner and patient, and importantly should empower the patient to be proactive in improving their health and well-being; that is, be involved in their self-care [12]. The notion of self-care was espoused in an Australian report, The State of Self Care in Australia which states that: “A healthy population is achieved through a functional relationship between active and informed individuals, health care services that empower and support people, and governments that invest in the capabilities of individual communities to look after their health” [13].

There is a growing interest in the field of oncology in relation to the integration of complementary therapies. Jentzsch and colleagues [14] concluded that in pancreatic ductal adenocarcinoma, an integrative treatment regimen combining first-line gemcitabine chemotherapy with two sub-groups of complementary medicines alternately in weekly cycles offers positive patient outcomes.

In this paper, we discuss key modifiable factors associated with cancer such as stress, nutrition, lack of physical activity, poor sleep and Vitamin D deficiency, including providing some of the evidence from an epidemiological research perspective of why these should be addressed. We will also briefly describe how these factors impact the pathomechanisms associated with cancer. An integrative approach that addresses these factors may contribute to better outcomes and better overall health of the patient. We examine some of the evidence that complementary medicines and approaches, including supplements, herbs and body therapies may be useful in an integrative approach to cancer treatment. Finally, we will offer our thoughts on how integrative oncology might be practised.

## 2. Addressing Modifiable Factors Associated with Cancer

Factors that can contribute to poor health are numerous; however, some key ones that are modifiable by individuals include stress, poor nutrition/diet, a lack of physical activity, poor sleep and inadequate levels of vitamin D, which have all been found to be associated with cancer [12]. These factors can contribute, at the very least, to a less than optimal quality of life. However, how do we relate these factors to what happens within the body when there is cancer?

The pathomechanism of cancer is complex. A seminal paper was published in 2000 on the “hallmarks of cancer” which set out an organizational framework of cellular properties uncovered during the transformation of normal cells to benign or malignant growths [15]. This was updated in 2011 with the inclusion of reprogramming of energy metabolism and evading immune destruction as additional hallmarks and the addition of two more enabling factors, tumor-promoting inflammation and genomic instability and mutation [16]. Then, in 2022 it was further updated to include phenotypic plasticity and disrupted differentiation as hallmark capabilities, and non-mutational epigenetic reprogramming and polymorphic microbiomes as enabling characteristics that can aid the acquisition of hallmark capabilities [17]. Kenny and colleagues [18], as well as many others (e.g., [16,17,19], draw attention to the importance of the tumor microenvironment and how it can influence the development and progression of tumors. The role of chronic low-grade inflammation in cancer is well established [17,20,21]. We can posit that factors such as stress and poor diet may contribute to chronic inflammation (discussed later) as part of Hanahan’s conceptual framework (the enabling factor of tumor-promoting inflammation).

The nervous system is also involved in cancer aetiology and pathogenesis [22,23]. Research indicates that tumour growth occurs in relation to the nervous system, indicating a functional role of nerves, neurons, neurites and neuroglia in tumorigenesis, discussed in depth by Baraldi and colleagues [22]. For example, tumour cells can release neurotrophins which stimulate adjacent neurites to grow into the tumour, and neurons can release neurotransmitters that initiate tumour cell migration [22]. Research also suggests sensory nerves may be able to regulate growth of tumours and metastasis by promoting or inhibiting immunosuppression [24]. The field of neuroimmunooncology is concerned with the interaction between the nervous system, immune system and cancer, and an effective approach to cancer may be one that targets the nervous system, and immune and/or genetic elements of the tumour micro/macro environments [22]. The concept of the neurobiology of cancer also recognizes the role of psychosocial factors in cancer [23].

Another conceptual framework, applied to nasopharygeal cancer (NPC) in a recent paper envisages NPC as a “spatiotemporal ‘unity of ecology and evolution’ disease: a multidimensional evolutionary adaptive pathological ecosystem” [25], describing how cancer tissues create a complex, spatially structured ecosystem of various cell types and essential stromal resources [25]. This framework could be extended to other forms of cancer to understand pathogenesis, as well as develop preventive strategies and therapeutic approaches (see [25]. Here, the concept of an ecosystem is applied at the tissue/molecular level to understand cancer pathogenesis, but this concept of an ecosystem could be extended outwards to a meta-level. That is, it could be used to describe the interdependence of humans and the environment in which we live and how, if this ecosystem becomes pathological, diseases like cancer can occur. The concept of environment can be broad, encompassing several dimensions such as the internal milieu within the body (i.e., internal environment, as described by Luo [25] and Hanahan [17]), the patient’s personal environment (e.g., immediate living and work environment), social/community environment and the greater environment (including climate, air quality, water quality, general standards of living, food supply and quality, and so on). All of these dimensions of “environment” are interdependent. The idea of a broader ecosystem is perhaps another way to understand how so many factors can contribute to cancer, and why the approach to its treatment should be multifactorial. This draws on one of the philosophies underpinning traditional Chinese medicine, as well as other systems of traditional medicine, considered to be of the “interdependence of man and environment” [26].

Whilst there are clearly many possible factors that can contribute to the development of cancer in an individual, we will focus on some key ones that are largely under the control of the patient, and for which doctors and oncologists may be able to offer assistance and/or advice.

In this section, we provide a rationale for why factors such as stress, poor sleep, poor diet, lack of physical activity and deficient Vitamin D levels should be addressed as part of an integrative approach to cancer management, drawing on just some of the evidence in the literature from epidemiological studies, as well as preclinical studies investigating mechanisms of action (though the latter will not be covered in depth due to the complexity of this area). Overall, we set out to stimulate thought on why such factors, those that patients can be empowered to address, ought to be considered by oncologists. In general, a healthy cancer patient is likely to do better than an unhealthy one.

### 2.1. Stress

The prevalence of psychological stress, anxiety and depression are major factors in cancer patients [27,28,29,30,31]. For example, the prevalence of depression may be anywhere from 25% to 66.7% [28,29,30], whilst the prevalence of anxiety has been found to be around 20% in one study [31].

Anxiety and depression are often comorbid [32] and both comorbid with other symptoms of cancer/cancer treatment, e.g., cancer-related fatigue [33], insomnia [34,35,36] and chronic pain [37,38]. Anxiety and depression can reduce quality of life and hinder cancer treatment, recovery and survival [39].

Some studies indicate that stressful life events can precede cancer [40,41] and that stress-related psychosocial factors are associated with higher cancer incidence and poorer survival [42]. Research suggests that depression may be associated with increased risk of cancer and may also predict cancer progression and decreased survival time [43,44,45,46].

**Figure 1:**
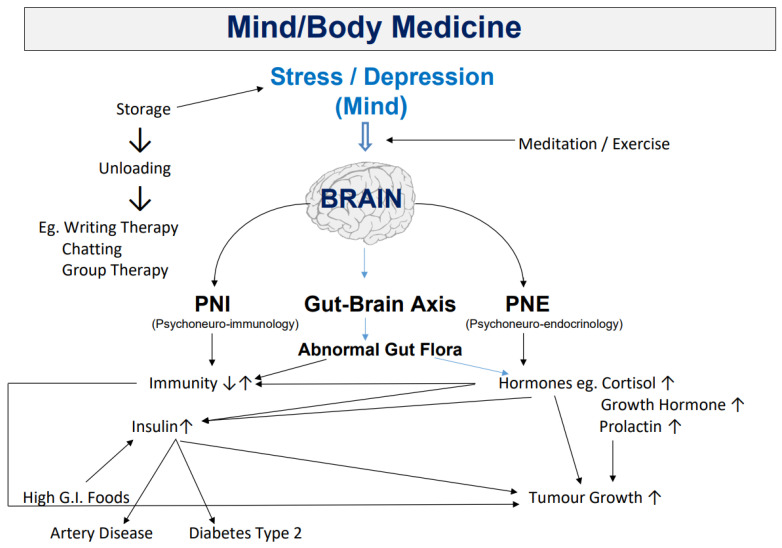
Pathomechanisms by which stress can contribute to cancer (figure reprinted with permission from [12]).

There is evidence that chronic stress can induce tumorigenesis and promote cancer development [22,47], including influencing steps or pathways involved in metastatic spread [48]. Research in the field of psychoneuroimmunology has demonstrated ways in which the nervous system exerts complex effects on anti-cancer immunity [23]. Stress can impact on the immune system and endocrine systems, as well as the gut–brain axis (leading to abnormal gut flora) and these can impact on immunity and hormones (such as cortisol, growth hormone and prolactin) to promote tumour growth, as set out in Figure 1 [12]. Mravec [23] explains that cancer is also able to manipulate the nervous system. For example, it can induce new sympathetic nerves to grow into tumour tissue and cause the trans-differentiation of a sensory neuronal phenotype to adrenergic to utilise the stimulatory effect for adrenergic signalling, to promote cancer growth and metastasis.

Neurotransmitters released from nerves innervating tumor tissues can affect the growth of tumours as well as metastasis [23], and neurotransmitters involved in stress reactions have been found to impair the function of several subsets of leukocytes [22]. As pointed out by Baraldi and colleagues [22], immune suppression by the nervous system due to chronic stress, anxiety or depression, can facilitate tumour development.

From this we can begin to see a convergence of the fields of psychoneuroimmunology and neuroimmunooncology that may further elucidate how stress, anxiety and depression are involved in cancer.

Pathomechanisms by which chronic stress promotes cancer development are described in more detail in Dai et al. [47] and include the production of stress hormones (via activation of the hypothalamic-pituitary-adrenal axis and sympathetic nervous system), which then promote tumorigenesis and cancer development via several mechanisms. These include increasing p53 degradation, inducing DNA damage accumulation, increasing inflammation, suppressing the immune system and its surveillance functions, and acting on tumour cells and stromal cells within the tumour microenvironment, facilitating tumour growth, invasion and metastasis [47].

Loneliness and social isolation can impact on cancer, upregulating genes associated with gene transcripts involved with tumour progression and in high-risk patients, increasing intra-tumoural norepinephrine [49]. In a study of 9247 women with breast cancer, those who were socially isolated women (small networks) were 1.43 times more likely to have a breast cancer recurrence, 1.64 times more likely to die from breast cancer and 1.69 more likely to die of any cause than more socially integrated women [50]. Social support can affect cancer survival favourably [51,52,53]; for example, having at least one confidant reduced seven-year mortality by 39% in women with breast cancer [52].

A sensitive person will be more susceptible to depression and stress, compared to an insensitive type of person who may not even be aware of these factors.

Research indicates that many different approaches to stress reduction can be useful and improve quality of life in cancer patients. For example, practising transcendental meditation for 8 years was associated with a 49% decrease in rate of mortality from cancer [54]. Examples are set out in Table 1.

The Ornish Lifestyle Program is a lifestyle-driven approach used initially to control coronary artery disease (CAD) and other chronic diseases. It promotes lifestyle changes including a whole foods, plant-based diet low in fat, high in vegetables, fruits, wholegrains, legumes and soy products, and supplementation of Fish Oil, Vitamin E, Vitamin C and Selenium; smoking cessation; moderate exercise 30 min per day for 6 days; stress management techniques including yoga, meditation and breathing techniques, and weekly psychosocial support group meetings [68]. Studies in men with prostate cancer who had chosen not to undergo orthodox treatment found that those assigned to the lifestyle changes achieved significantly greater decreases in prostate-specific antigen (PSA) compared with those in the usual care control group [68] and had positive changes in gene expression over three months [69].

### 2.2. Disturbed Sleep

The prevalence of sleep disturbance in newly diagnosed or recently treated cancer patients is estimated to be 20–75% [70,71,72] and insomnia is found in 23–44% of patients 2–5 years after treatment for cancer [73,74]. Insomnia and sleep disturbances can lead to fatigue, mood disturbance, and contribute to immune suppression, affect quality of life, and may negatively impact on the disease course [75]. The National Cancer Institute [76] advises that up to 50% of cancer patients have sleep-related problems during treatment; these may be due to side effects of the treatment or pharmaceuticals, stress, long hospital stays and other factors.

Insomnia may be a risk factor for cancer, though as pointed out by Shi and colleagues [77], results of epidemiological studies have been somewhat equivocal as to whether there is a relationship between insomnia and cancer, with several suggesting a higher risk of cancer associated with insomnia [78,79,80,81] and others suggesting no association [82,83,84,85]. A meta-analysis conducted by Shi et al. [77] of these 8 studies found there was a modest 24% increased risk of cancer in those with insomnia (compared to those without insomnia). A recent review by Berisha and colleagues [86] indicates that chronic disruption of sleep/wake states prior to disease onset is associated with an increased risk of some cancers, e.g., breast, and that sleep disruption after cancer onset is often associated with poorer outcomes.

How disturbed sleep contributes to the development of cancer is complex. For example, mice experiments have shown that sleep characterized by frequent awakenings can speed the growth of cancer, increase tumor aggressiveness and depress immunity [87]. In sleep deprived mice, tumor associated macrophages (which can contribute to cancer progression by releasing different chemicals involved in tumour growth and invasion) were found to be more numerous and distributed closer to tumour capsules (compared with control mice) [87]. Sleep deprivation has been found to affect hundreds of genes related to circadian rhythms, metabolism, inflammation, immune response and stress [88]. As described by Shi et al. [77], other potential mechanisms of action proposed to explain how insomnia may contribute to cancer include dysregulation of melatonin [89] and circadian rhythm/chrono- disruptions (which can affect rhythmicity in neuroendocrine and immune parameters) [90], dysregulation of genes involved in tumour suppression [91], involvement of the oestrogen-signalling pathway [92], impaired immune function [93] and inflammation [94]. See Berisha et al. [86] and Shi et al. [77] for further discussion.

Approaches to improving sleep are many and include: general sleep hygiene guidelines, diet, exercise, stress reduction (e.g., relaxation/meditation/yoga), cognitive behavioural therapy, and supplements/herbs [12], including medicinal cannabis (discussed later). The US National Sleep Foundation’s 2013 Sleep in America Poll: Exercise and Sleep clearly shows beneficial effects of exercise on sleep, in particular, morning vigorous exercise [95].

Whilst it is beyond the scope of this paper to go into detail about different evidence-based complementary medicine approaches to each of the factors outlined above, readers are directed to any number of resources for further information, for example O’Brien and Sali [12] or Phelps [96]. In the following section, we will focus on examples of research into supplements and herbal medicines.

### 2.3. Diet and Nutrition

The link between diet and cancer has been revealed by the large variation in cancer rates between countries, as well as correlations with diet and observations of changes in cancer rates with migration [97,98]. Poor diet can lead to overweight and obesity, risk factors for cancer [99]: compared with those with a healthy weight, those with overweight or obesity have a greater risk of at least 13 types of cancer [100]. A 2019 study found that globally, excess body weight accounted for almost 4% of all cancers [101].

On the other hand, there are many studies which indicate that the adoption of particular diets such as the Mediterranean Diet are associated with reduced cancer incidence [102] and mortality [103].

Chronic, low-grade inflammation underpins many chronic illnesses such as cancer, as well as cardiovascular disease and type 2 diabetes, evidenced by elevations in levels of inflammatory biomarkers (e.g., C-reactive protein, interleukin 6 and 18, adhesion molecules (e.g., E selectin, intercellular adhesion molecule 1), vascular cell adhesion protein 1 and fibrinogen [104]. Diets high in sugar, saturated and trans-fatty acids and refined starches, and low in antioxidants, omega-3 polyunsaturated fatty acids (PUFAs) and fiber (e.g., from fruits, vegetables, whole grains) are proinflammatory and can activate the innate immune system, probably via increased production of proinflammatory cytokines and reduced production of anti-inflammatory cytokines [104]. The typical western diet has a ratio of the (more pro-inflammatory) omega 6 PUFAs to (anti-inflammatory) omega 3 PUFAs of between 10: and 30:1 [105,106,107], yet we evolved on a diet where this ratio was probably around 1:1 [105].

The Mediterranean Diet is characterized by high consumption of olive oil, fruits, vegetables, cereals (whole grains), legumes, seeds and nuts, moderate amounts of fish (a source of Omega 3 PUFAs), shellfish, white meat, eggs, fermented dairy products (e.g., cheese, yoghurt), and small amounts of red meat, processed meat and foods high in sugar, and includes the consumption of wine, especially red wine [104]. This diet has a high amount of omega 3 PUFAs (from fish and plants) and a low omega 6: omega 3 ratio of around 2:1 to 1:1 [104]. It has phytochemicals such as vitamin C, vitamin E, folate, carotenoids and polyphenols which have antioxidant and anti-inflammatory properties [104]. High dietary intake of antioxidants, including polyphenols associated with diets such as the Mediterranean Diet, may inhibit several cancer-related biological pathways, including reducing inflammation. Antioxidants play a role in cell differentiation and proliferation, and in synthesis and DNA repair by inhibiting production of carcinogenic chemicals endogenously, and reducing formation of adducts in DNA [104]. Omega 3 PUFAs can impact several of the cancer pathways including cell proliferation, cell survival (e.g., promoting apoptosis), inflammation, angiogenesis, metastasis and epigenetic abnormalities [108]. In animal models of breast cancer, diets high in extra virgin olive oil were found to induce different molecular changes in tumours, including in the activity of signalling molecules and gene expression, changes which induced lower proliferation, higher apoptosis and lower DNA damage compared with other diets [109].

Many studies have found that diets high in vegetables and fruit are protective against cancer and may play a role in prevention of disease recurrence [98,110,111,112,113,114,115,116]. Olive oil is also a key protective component compared to unstable vegetable oils used in the western diet. A systematic review found that high olive oil intake was associated with significant reduction in the risk of several cancers [117]. Yet, the Mediterranean Diet is not perfect; it includes preserved meats, wine and other foods which are not necessarily healthy. What might be protective may be the context of taking meals: meals are usually a social, family event. The social context of eating may be protective in itself [12]. It is very likely that the cultural aspects play a key role.

Diets that are high in meat and fat and low in dietary fibre are associated with greater risk of colorectal cancer (CRC) [118]. There is increasing evidence that CRC is occurring more frequently in younger people, with those consuming large amounts of deep-fried foods, refined foods, sugary drinks and desserts, and those on high fat diets and with low fibre and folate consumption being at higher risk [110].

Recent reviews of the role of dietary fibre reinforce the importance of these types of nutrients in the protection of cancer and other diseases [119,120]. Dietary fibre can influence metabolism in particular glucose and subsequent insulin, as well as have a major influence on microbiome [121,122]. The microbiota plays a role in bile acid metabolism, and epidemiological evidence supports the role of diet in modifying the composition and levels of bile acids, which in turn can modify the risk of colorectal cancer at the population level [118].

### 2.4. Inadequate Vitamin D

Research suggests a link between low Vitamin D levels and cancer. Systematic reviews have found associations between Vitamin D levels and risk of cancer, with higher levels being associated with lower risk [123,124,125,126]. In those with cancer, lower vitamin D levels are associated with greater cancer-related and all-cause mortality [127,128,129,130,131]. Inverse relationships are demonstrated between solar UVB and incidence and/or mortality rates for 22 types of cancer [132].

Vitamin D receptors are widely distributed in the body, including in immune response cells, supporting its role in homeostasis [133]. Vitamin D appears to play a role in protecting against cancer via several mechanisms, including inhibiting initiators of cellular angiogenesis (in various cancer cell lines), promoting antioxidant responses, inhibiting cell proliferation, stimulating DNA repair, suppressing metastasis and regulating autophagy [132,133]. Thus, a deficiency in Vitamin D could be relevant in the development of cancer [12].

Experimental studies indicate vitamin D has anti-neoplastic activity (apoptosis was induced by a metabolite of vitamin D in colorectal adenoma and carcinoma cells [134] and anti-proliferative activity [135]. Other preclinical studies (laboratory, animal) have found that vitamin D can inhibit carcinogenesis, slow tumour progression, inhibit cancer cell proliferation, promote apoptosis and is anti-inflammatory and anti-angiogenic [136].

Several studies have investigated the effect of vitamin D supplementation in cancer patients, with mixed results. For example, in a randomised controlled trial (RCT) of patients with digestive tract cancers, the AMATERASU trial in Japan, supplementation with 2000 IU vitamin D daily was not associated with any significant improvement in relapse-free survival at 5 years compared with placebo [137], though a post hoc age-adjusted analysis of the data actually showed a statistically significant benefit associated with supplementation (relapse-free survival HR, 0.66; 95% CI, 0.43–0.99) [136,137]. In a small study of 139 patients with advanced or metastatic colorectal cancer, high dose vitamin D (8000 IU daily for two weeks, and 4000 IU thereafter) was compared with standard dose vitamin D (400 IU daily) as an adjunct to standard chemotherapy. The high dose vitamin D was associated with a non-significant improvement in progression-free survival (13 months versus 11 months, *p* = 0.07) and significantly lower risk of progression-free survival or death (HR, 0.74; *p*  =  0.02); the latter effect was greater in those with lower BMI, prompting the researchers to opine that the results warranted larger scale studies [138].

A meta-analysis of RCTs found that over 2–7 years of supplementation with vitamin D had little effect on total cancer incidence (4 RCTs, n = 4333 participants, 400–1100 IU daily) but it was associated with significantly reduced total cancer mortality (3 RCTs, RR 0.88, 95% CI 0.78–0.98) [139]. In the large (n = 25,871) VITAL study in the US, vitamin D supplementation was not associated with a lower incidence of invasive cancer compared with placebo [140]; however, a secondary analysis indicated vitamin D supplementation was associated with a reduced risk of advanced (metastatic or fatal) cancer, with the strongest reduction in risk in those with normal weight [136].

Sunlight exposure is the best way to increase vitamin D, rather than via foods; however, when sunlight exposure is low, supplementation may be needed [12].

### 2.5. Inadequate Physical Activity

A meta-analysis of 17 prospective studies (total of 857,581 participants) found that sedentary behaviour significantly increased the risk of cancer by 20% [141]. Sedentary behaviour is associated with a higher risk of many cancers, for example endometrial [141,142], colorectal and its recurrence [141,142,143,144], breast cancer [141] and its recurrence [145], lung cancer [141], and prostate and ovarian cancer [142]. In addition, low levels of physical activity are associated with increased risk of all-cause and disease-specific mortality in cancer survivors [146]. Sedentary activity can be also associated with obesity, a risk factor for cancer [147], and there is evidence that weight loss can reduce risk of several cancers [148].

Positive news is that scientific evidence suggests that physical activity is protective against cancer [149,150,151]. In a meta-analysis which pooled the results of 12 prospective US and European cohorts (a total of 1.44 million subjects), in comparison with low levels of leisure-time physical activity, high levels of physical activity were significantly associated with lower risks of 13 cancers (10 of these associations remained statistically significant after taking body mass index into account) [151]. Exercise has been found to reduce risk factors for cancer such as obesity [152] and inflammation [153,154].

There are many benefits associated with exercising during and/or after cancer treatment [12], including helping protect against cancer-related fatigue [155] and improving health related outcomes in cancer survivors [156]. Physical activity after cancer diagnosis can reduce risk of all-cause death, as well as cancer-specific mortality [146,150,157,158]. Australian research found that in men with prostate cancer, exercise promoted the production of myokines (cytokines produced by muscle and secreted into the bloodstream) [159] which may be involved in exercise-induced tumour suppression [159,160]. Other benefits of exercise in cancer patients include improvements or preservation of muscle mass, strength and power, reduction in symptoms and side effects (including nausea, fatigue and pain), increased cardiorespiratory fitness, increased physical function, increased immune function, increased chemotherapy completion rates, reduced treatment-related side effects, improved curative effects of other treatments, better body image and self-esteem, decreased psychological and emotional distress, reduced depression and anxiety and shorter length of hospitalisation [161,162].

According to Friedenreich and colleagues [147], the molecular mechanisms by which sedentary lifestyles, lack of physical activity and obesity may contribute to cancer include effects on endogenous sex steroids and metabolic hormones, insulin sensitivity and chronic inflammation. Meanwhile, the mechanisms by which physical activity may reduce cancer risk are many and include decreasing systemic inflammation, hyperinsulinemia, insulin-like growth factor (IGF-I), sex hormones, pro-inflammatory leptin plus other cytokines associated with obesity, significantly increasing levels of (the anti-inflammatory) adipoleptin, improving immune function and the diversity and composition of the gut microbiome [149]. Other mechanisms include regulating cancer cell metabolism, regulating the immune environment, regulating growth factor secretion, targeting Akt and mTOR pathways, regulating skeletal muscle IL-6, and improving mitochondrial function which can inhibit cancer cell proliferation as well as apoptosis, though only moderate intensity exercise appears to exert significant influence on cancer cell proliferation and apoptosis [162]. During physical activity, contracting skeletal muscles release IL-6, a myokine, which exerts anti-inflammatory effects in other organs via an inflammatory (TNF-α) independent pathway, and the release of IL-6 induces increased anti-inflammatory interleukins IL-1ra and IL-10 [149]. Anti-inflammatory effects can occur via the reduction in visceral and body fat in addition to the anti-inflammatory mileu created through myokine release [149]. This helps explain how the anti-inflammatory effects of exercise are protective against systemic low-grade inflammation that underpins chronic illnesses such as cancer [149]. See Wang and Zhou [162] and Jurdana [149] for more information.

## 3. Complementary Medicines and Approaches in the Integrative Management of Cancer

Complementary medicine/treatment approaches may have beneficial effects in addressing many of the symptoms and signs associated with cancer and its orthodox treatment, as well as potentially addressing the pathomechanisms associated with cancer.

In this section, we describe just some of the evidence that complementary medicines or treatment approaches might be useful adjuncts in an integrative approach to cancer management.

### 3.1. Dietary Supplements

When a person does not have a sufficient variety of foods—particularly vegetables—in their diet, they can become deficient in vitamins and trace elements. Adequate micronutrients are essential for the proper functioning of the immune system and deficiency suppresses immunity [163]. Dietary supplements can help to ensure a person is as healthy as possible, where dietary sources of vitamins and minerals might be deficient [12]. For example, selenium has been primarily considered to have antioxidant, anti-inflammatory and anti-viral activity, but emerging evidence suggests a role in several of the pathways involved in cancer, including cell proliferation, migration, invasion and angiogenesis [164].

Another example of a type of supplement that can play an important role in an integrative approach to cancer is probiotics. The gut microbiome plays a critical role in the functioning of the immune system, influencing inflammation as well as the nervous system [165], and there is evidence that the bacterial microbiota plays a key role in carcinogenesis [166]. Stress can negatively impact the gut microbiome and chemotherapy and other drugs can damage it [12,167,168]. Chemotherapy can adversely have an impact on the gut microbiota, causing dysbiosis and altering its physiological and psychological functions [168]. Cancer treatments can alter the oral and gut microbiota and cause intestinal dysfunction, contributing to the pathogenesis of oral mucositis [167].

Other important supplements for cancer patients include: fish oil, vitamin C, co-enzyme Q10, magnesium, lycopene, vitamin E and vitamin B3 (this last one is in relation to skin cancer) [12,169].

There is evidence that antioxidants slow cancer progression and prevent its spread [170,171,172,173,174,175]. It is worth addressing the long-standing misperception that antioxidants might reduce the effectiveness of chemotherapy and radiation by reducing the potency of free radicals needed to kill cells [176]. Overall, research indicates that antioxidants in general (including low dose dietary forms and high dose IV forms) provide many benefits, do not reduce the efficacy of chemotherapy or radiation therapy, and can increase the effectiveness of conventional cancer therapeutic agents and decrease adverse effects [170,176,177,178,179,180]. For a concise explanation see Gonzales et al. [176]. A systematic review of 19 clinical trials investigating the use of antioxidants including glutathione (7), melatonin (4), Vitamin A (2), Vitamin C (1), Vitamin E (1), ellagic acid (1), N-acetylcysteine (1), and an antioxidant mixture (1), found that none of the studies reported evidence of significant decreases in chemotherapy efficacy due to antioxidant supplementation during chemotherapy [177]. Another systematic review also concluded that no trial reported a significant decrease in treatment efficacy with antioxidant use, and when antioxidants are included in a cancer patient’s therapeutic regime, there are several benefits, including reduced toxicity, improved treatment outcomes, increased survival times, increased tumour responses, and increased adherence to chemotherapy regimes [178].

### 3.2. Intravenous Supplements

The following supplements can be given at a much higher dose intravenously than orally: vitamin C, glutathione and A-Lipoic Acids. There is increasing evidence that high dose IV vitamin C has potential as a strong anti-cancer agent, with early phase clinical studies demonstrating efficacy in eradicating various types of cancer cells, as well as its safety [181].

The anti-cancer mechanisms of high doses of ascorbic acid (ascorbate) include pro-oxidant cytotoxic activity, inhibiting cell proliferation via inhibiting prostaglandins (2 series), inhibiting angiogenesis, anti-cancer epigenetic regulation, immune regulation, reversing epithelial-to-mesenchymal transition, inhibiting hypoxia and oncogenic kinase signalling and boosting the immune response [176,181,182]. High dose IV vitamin C is able to act synergistically with many standard chemotherapy drugs and mitigate their toxic side effects [181]. Adjunct IV Vitamin C administration for at least four weeks in breast cancer (stages IIa to IIIb) survivors undergoing chemotherapy or radiation therapy was associated with a significant reduction in side effects induced by the disease or the treatment, including nausea, loss of appetite, fatigue, depression, sleep disorders, dizziness and haemorrhagic diathesis compared to women without this adjunct treatment. There were no documented side effects of the IV vitamin C [183]. An important point in relation to surgery is that anaesthesia and surgery deplete vitamin C; humans cannot produce vitamin C, which is essential for brain function and wound healing [184]. Further clinical studies need to be carried out to evaluate the role of high dose IV Vitamin C for post-surgery treatment, including in cancer patients.

Vitamin C is relatively non-toxic at high levels. However, patients with glucose-6-phosphate deficiency may be at risk of developing haemolysis when given high doses of Vitamin C and hence patients need to be screened prior to high dose therapy.

### 3.3. Herbal Medicine

Many herbs and spices have been found to have anti-cancer properties in preclinical studies. Here are just a few examples.

Curcumin, a component of the well-known Asian spice turmeric (Curcuma longa), has anti-inflammatory, antiseptic, analgesic, antioxidant and anti-proliferative activity [185,186]. Curcumin has several anti-cancer actions via its effect on pathways involved in cell cycle regulation, apoptosis, mutagenesis, oncogene expression, tumorigenesis, and metastasis [186]. Curcumin can inhibit lung cell proliferation [187], inhibit prostate cancer cell growth via inhibition of androgen receptor pathways [188], inhibit prostate cancer bone metastasis [189], and inhibit epithelial mesenchymal transition and invasion induced by cancer-associated fibroblasts in prostate cells [190]. It can modulate many molecular targets, including transcription factors, growth factors and their receptors, cell adhesion molecules, enzymes, cytokines and genes involved in tumour growth, angiogenesis and metastasis [190,191]. Curcumin can modulate the growth of tumour cells through several cell signalling pathways, including cell proliferation, cell survival, caspas, tumour suppressor (p53, p21), death receptor, protein kinase and mitochondrial pathways [185]. Research in animals demonstrated its ability to inhibit tumour initiation and tumour promotion [185,192]. In humans, curcumin has also been found to be effective in treating depression and anxiety [193,194], which is relevant to cancer.

The herb cannabis sativa may be useful in the alleviation of several symptoms and signs associated with cancer and its orthodox treatment. These include cancer-related pain, cachexia, anxiety, depression, oral mucositis, chemotherapy-induced nausea and vomiting and sleep disorders [1]. Importantly, components of cannabis such as cannabidiol (CBD) may be protective against chemotherapy drug toxicity. CBD has demonstrated cardio-protective effects in animals against doxorubicin-induced cardiac injury and nephron-protective effects against cisplatin [195,196]. Mice studies found that CBD is protective against paclitaxel-induced neurotoxicity [197], though caution is advised with respect to immunotherapy drugs [198]. See O’Brien [1].

The Chinese herb Ganoderma lucidum (*G. lucidum*, Chinese herb Ling Zhi) has been shown to have anti-cancer properties. Preclinical research has demonstrated several anti-cancer mechanisms, including immunomodulation, inducing cell-cycle arrest and apotosis (several types of human tumour cells) and inhibiting cell adhesion, invasion and migration and angiogenic factors [199,200]. In animal experiments, a triterpene rich extract of *G. lucidum* was found to suppress prostate growth induced by testosterone [201], and one of its active constituents Ganoderol B was found to bind to the androgen receptor and inhibit 5α-reductase, suppress androgen-induced LNCaP cell growth, and downregulate Prostate-Specific Antigen (PSA) [202]. In a study of patients with advanced-stage cancers, supplementation with Ganopoly, the polysaccharides fractions extracted from *G. lucidum*, for 12 weeks resulted in significant enhancement of cellular immunity (elevated IL2, IL6 and interferon γ in 80% of patients [203]. A systematic review found that patients who had taken *G. lucidum* as an adjunct therapy with chemo/radiotherapy were more likely to respond positively compared to the orthodox treatment alone (RR 1.50; 95% CI 0.90–2.51, *p* = 0.02) [204]. Cao and colleagues [199] provide a comprehensive explanation of the research evidence for the anti-cancer actions of *G. lucidum*.

Chinese herbal medicine (CHM) typically uses combinations of herbs rather than single herbs. CHM may be a useful adjunct to orthodox treatment, enhancing the tumour response to chemotherapy and reducing toxicity [205]. It can also be useful in the treatment of many side effects of cancer and its orthodox treatment including, but not limited to the following: xerostomia following radiation therapy; nausea and vomiting (in particular associated with chemotherapy), other digestive problems (e.g., diarrhoea, constipation), anorexia, polyneuropathy, hot flushes, anxiety, depression, insomnia, pain and cancer-related fatigue [205,206,207,208].

### 3.4. Acupuncture

Evidence from randomised controlled trials suggests acupuncture is useful in the treatment of many symptoms and signs associated with cancer and its treatment, including chemotherapy-related nausea and vomiting, cancer fatigue, neutropenia, cancer-related pain and xerostomia [209,210,211,212,213,214]. For example, in a randomised controlled trial of 86 nasopharyngeal cancer patients, acupuncture was associated with positive effects on xerostomia as early as 3 weeks, with significantly greater saliva flow at week 7 and at the 6 months follow-up (*p* < 0.003). At the 6 months follow-up, 24% acupuncture group had xerostomia compared with 63% controls [210].

### 3.5. Massage

Scientific studies have demonstrated that oncology massage can reduce symptoms, including stress, pain, anxiety, depression, nausea and fatigue in those who have had surgery or chemotherapy for cancer [215]. Other benefits for cancer patients include improving coping, improvements in fatigue, pain and stress, enhanced quality of life, as well as improved immunity (lymphocyte count) [216,217,218].

## 4. Repurposing of Current Drugs and Their Influence on Metabolic Blockade

A potential source of new treatment options for cancer patients is the repurposing of existing non-cancer drugs. Off-label use of drugs is common in general practice and paediatrics where up to 10% of drugs are used off-label [219]. In particular, in oncology the practice of off-label use of drugs is even more common; in some studies up to 71% of adult cancer patients have been prescribed off-label drugs [220]. As the development of new cancer drugs is a challenging and costly endeavor, drug repurposing is regarded as an alternative to potentially accelerate this process.

There are several drugs which have been repurposed for the treatment of cancer. There is evidence that cancer cells share the same metabolic profile as trophoblastic cells, but different from adult somatic cells, e.g., use of glutamine, aerobic glycolysis [221]. Therefore, using off-label medications to block cancer metabolic pathways is a sound strategy in conjunction with standard care as its effect on non-cancer cells are limited and known to be safe in the doses used regularly for its on-label indications.

For example, studies have demonstrated evidence suggesting a potential role for metformin in cancer therapy. Preclinical studies have demonstrated several anti-cancer molecular mechanisms of metformin including mTOR inhibition, cytotoxic effects and immunomodulation. Epidemiologic data have demonstrated decreased cancer incidence and mortality in patients taking metformin. Several clinical trials, focused on evaluation of metformin as an anti-cancer agent are presently underway [222]. Metformin blunts the Warburg effect and consequently downregulates the growth of cancer stem cells. It has been suggested that metformin may well be used as a radiation sensitizer or an immunotherapy drug, in addition to a direct anti-proliferative agent for the treatment of cancer [223]. The use of metformin in diabetic patients diagnosed with cancer has been associated with a decrease in risk of cancer mortality [224].

Naltrexone (NTX) is an opioid antagonist traditionally used as a treatment for alcohol and opioid use disorders, but various studies have documented its involvement in cancer progression, when used as low dose treatment. Further evidence is necessary to demonstrate its efficacy and its mechanisms of action [225].

Melatonin has been used at high dosage, as an adjuvant in radiotherapy for radio-protection and radio-sensitization. The molecular mechanisms for radio-protection and radio-sensitizer effects of melatonin have been investigated. High dosages such as 60 mg, four times per day have been used to induce oncostasis and also reduce radiation side effects [226].

Dichloroacetate (DCA) works by turning on the natural cell suicide system, apoptosis, which is suppressed in cancerous cells, thus allowing their destruction. DCA does not poison the cells like cytotoxic chemotherapy drugs; it interferes with the utilisation of glucose by the cancer cell, leading to its starvation. It can improve the sensitivity to various drugs or radiotherapy, leading to apoptosis [227].

Sodium Phenylbutyrate (PB), a drug typically used in urea cycle disorders, is another useful drug that helps reduce serum levels of glutamine, which most cancers are dependent on without affecting normal cells. Cancer cells are well known to be addicted to glutamine as its major fuel source apart from glucose [228]. Lowering serum levels with phenylbutyrate helps to deprive one of their main sources of energy. PB also helps to induce cell differentiation, which makes for a less aggressive phenotype, a particular useful strategy in cancer therapy [229]. It is also a histone deacetylase inhibitor, which helps to reduce gene expression in cancer cells [230].

Several other drugs have been explored because of their repurposing potential, including Sodium Phenylbutyrate; Atorvastatin; Dipyridamole; Doxycycline and Mebendazole.

However, although repurposing drugs is an appealing strategy, this is no substitute for randomized clinical trials to determine efficacy and the populations most likely to benefit from an intervention.

## 5. Other Therapies for Cancer Treatment

There are other therapies which may be useful in the treatment of cancer. The National Institute of Integrative Medicine in Australia has been investigating the role of Photodynamic Therapy for the treatment of prostate cancer with promising preliminary results. The laser is delivered to the prostate trans-rectal and trans-urethral (see Meade et al. [231]). Hyperthermia and hyperbaric therapy are increasingly being used in the treatment of cancer, but due to limited capacity within this paper, details are not included.

## 6. Mitigating Risk in an Integrative Approach to Cancer Treatment

There are naturally concerns about whether any nutritional supplements and herbal medicines could adversely interact or interfere with orthodox cancer treatment. The potential for adverse interactions between certain drugs and certain forms of complementary medicines is well documented in the literature [232,233,234,235]. On the other hand, many complementary medicines have benefits in combination with orthodox cancer treatment (e.g., [1,137]. For example, the use of a common Chinese herbal formula called Jia Wei Xiao Yao San with Tamoxifam reduced the risk of subsequent endometrial cancer in female breast cancer patients [236].

There are drug-supplement/herb databases that set out the scientific evidence in relation to potential interactions, such as the IMGateway, an online portal for healthcare practitioners which is being adapted for consumer use [6]. Use of such evidence-based portals by practitioners can reduce potential for adverse interactions and provide a level of confidence for both patient and practitioner.

## 7. How to Practise Integrative Oncology

It is not possible to be an expert in oncology as well as nutritional medicine, stress reduction techniques, herbal medicine and so on. However, doctors can inquire about those factors that can impact on cancer patients (covered earlier in the paper) and facilitate referral to other healthcare practitioners capable of assisting. A team approach that integrates input from clinicians with different types of expertise is ideal, and often the general practitioner has an important role to play here. Remember, research indicates that a significant proportion of patients (42% in one US study) do not speak to their doctor about their complementary medicine use, often fearing disapproval [237]. Those patients who want to use complementary medicine approaches are likely to do so with or without your approval. Therefore, it is important to keep an open mind, and read and critique the scientific evidence available on complementary medicine approaches—there is much more research evidence than many think.

Deciding which complementary medicines or approaches to integrate with standard therapy can be done systematically and rationally, as exemplified by Jentzsch and colleagues [14]. In their paper, they set out an evidence-based approach to choosing which complementary medicines/therapies/approaches should be combined with gemcitabine in the treatment of patients with PDAC. They firstly divided the various complementary measures into three groups: dietary factors, nutraceutical agents and lifestyle. They then looked at the available evidence in relation to specific dietary and nutraceutical agents, considering clinical trials, meta-analyses, in vivo tests and in vitro studies. From this they were able to identify 9 agents: 6 dietary (Vitamins A, C, D and E, genistein and curcumin) and 3 nutraceutical compounds (propolis, triptolide and cannabidiol) that they deemed acceptable due to the available evidence base, for integration with gemcitabine chemotherapy. They propose an integrative treatment regimen combining gemcitabine chemotherapy with the two sub-groups of complementary agents alternately in weekly cycles could then be applied, with the ability to modify this protocol for poor responders or “super responders”. The judicious combining of particular complementary agents took into consideration their main mechanisms of action in relation to particular hallmarks of cancer [14]. This methodology could be readily adapted to other types of cancer and is an example of a systematic, rational approach to considering what types of complementary medicines might be safely combined with standard oncology treatment, taking into consideration safety issues such as potential interactions.

## 8. Conclusions

An integrative approach to cancer treatment uses the best of evidence-based conventional and complementary medicine approaches. It empowers the patient to be proactive in improving their overall health and addressing many of the factors associated with cancer, as well as symptoms and signs associated with cancer and its orthodox treatment. Helping the cancer patient address factors that are largely within their control such as stress, poor nutrition, poor sleep, vitamin D deficiency and lack of physical exercise may result in a better quality of life and better outcomes. There is much scientific research in the literature—preclinical and clinical—indicating that many forms of complementary medicine are effective in alleviating many of the symptoms/signs associated with cancer and its treatment, and may potentially also address the pathomechanisms underpinning various cancers. Cancer patients are using complementary medicines. By becoming better informed, doctors are in a more nuanced position to make recommendations to patients, as well as refer to a wider network of healthcare professionals as part of a team approach to cancer care.

## Figures and Tables

**Table 1:** Examples of stress reduction therapies useful for cancer patients.

Type of Therapy	Some Key Benefits	References
Meditation	Significantly less tumours compared with non-meditators; fewer admissions for cancer; decreased mortality from cancer; cancer regression	[54,55,56]
Tai Chi and Qi Gong	Positive effects on cancer-specific quality of life, as well as fatigue, anxiety, immune function and cortisol levels	[57]
Pet therapy	Significant improvements in social and emotional wellbeing despite high symptom burden and expected decreases in physical and functional wellbeing associated with radiation-chemotherapy treatment [58]; pet therapy during chemotherapy associated with significant decrease in depression and increase in oxygen saturation [44]	[58,59]
Art therapy	Decreased anxiety, depression and pain in patients with cancer; decreasing anxiety in those receiving cancer therapy; improvement in wellbeing	[60,61,62,63,64]
Music therapy	Reduction of anxiety, pain, fatigue and improved mood and quality of life	[65,66]
Writing therapy	Improved quality of life	[67]

## Data Availability

No new data were created or analyzed in this study. Data sharing is not applicable to this article.
